# MYC inhibitors in multiple myeloma

**DOI:** 10.20517/cdr.2021.55

**Published:** 2021-08-13

**Authors:** Sandra Martínez-Martín, Laura Soucek

**Affiliations:** ^1^Preclinical & Translational Research Program, Vall d’Hebron Institute of Oncology (VHIO), Vall d’Hebron Barcelona Hospital Campus, Barcelona 08035, Spain.; ^2^Peptomyc S.L., Vall d’Hebron Barcelona Hospital Campus, Barcelona 08035, Spain.; ^3^Department of Biochemistry and Molecular Biology, Universitat Autònoma de Barcelona, Bellaterra 08193, Spain.; ^4^Institució Catalana de Recerca i Estudis Avançats (ICREA), Barcelona 08010, Spain.

**Keywords:** MYC inhibition, multiple myeloma, undruggable target, targeted therapies, transcription factor, epigenetics, MYC downregulation

## Abstract

The importance of MYC function in cancer was discovered in the late 1970s when the sequence of the avian retrovirus that causes myelocytic leukemia was identified. Since then, over 40 years of unceasing research have highlighted the significance of this protein in malignant transformation, especially in hematologic diseases. Indeed, some of the earliest connections among the higher expression of proto-oncogenes (such as *MYC*), genetic rearrangements and their relation to cancer development were made in Burkitt lymphoma, chronic myeloid leukemia and mouse plasmacytomas. Multiple myeloma (MM), in particular, is a plasma cell malignancy strictly associated with MYC deregulation, suggesting that therapeutic strategies against it would be beneficial in treating this disease. However, targeting MYC was - and, somehow, still is - challenging due to its unique properties: lack of defined three-dimensional structure, nuclear localization and absence of a targetable enzymatic pocket. Despite these difficulties, however, many studies have shown the potential therapeutic impact of direct or indirect MYC inhibition. Different molecules have been tested, in fact, in the context of MM. In this review, we summarize the current status of the different compounds, including the results of their clinical testing, and propose to continue with the efforts to identify, repurpose, redesign or improve drug candidates to combine them with standard of care therapies to overcome resistance and enable better management of myeloma treatment.

## INTRODUCTION

### Multiple myeloma

Multiple myeloma (MM), although a rare disease, is the second most common blood cancer^[1]^, with over 54,000 estimated new cases in Europe^[2]^ and 176,000 worldwide in 2020^[3]^. It is a neoplasm originating from the clonal expansion of plasma cells in the bone marrow (BM)^[4]^.

**Figure 1:**
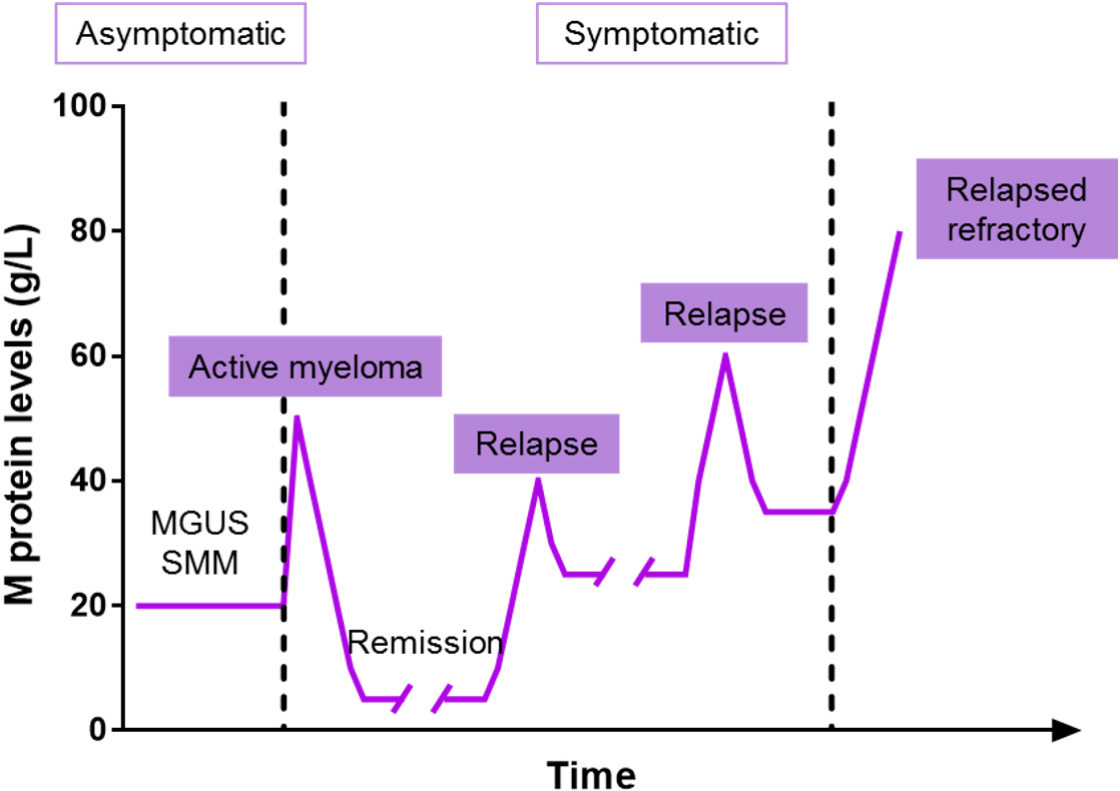
The response-relapse pattern in multiple myeloma patients. Monoclonal gammopathies that undergo malignant transformation are likely to respond initially to the therapy and enter in remission. However, the disease eventually relapses, and the response becomes less durable until resistance appears, resulting in relapsed refractory myeloma. Figure adapted from^[6]^. MGUS: Monoclonal gammopathy of undetermined significance; SMM: smoldering multiple myeloma.

Even if recent advances in medicine have quadrupled MM patient survival in the last 40 years (from 6% to 33% for ten or more years), it remains virtually incurable, as relapse rates are as high as 90%^[5]^. Indeed, the disease adopts a cyclical pattern of response to therapy and remission followed by disease progression or reappearance^[6]^, as depicted in Figure 1.

Importantly, due to the progressive aging of the population and the fact that the peak rate of MM cases is at 85-89 years^[7,8]^, MM’s incidence trend over time has increased by 32% since the early 1990s and keeps growing. In fact, estimates predict that the number of incident myeloma cases will almost double by 2040^[9]^.

Myelomagenesis is a complex process that requires various driver genetic alterations to collude with each other, resulting in the development and progression of MM^[10]^. A benign and asymptomatic condition, termed monoclonal gammopathy of undetermined significance, precedes it and can evolve into another asymptomatic disorder, also classified among monoclonal gammopathies, termed smoldering multiple myeloma (SMM). All these diseases are characterized by the invasion of proliferating plasma cells in the BM and the secretion of monoclonal proteins referred to as M protein or M spike, present in large amounts in the blood and urine and used for the disease diagnosis^[11-13]^. This protein is also known as paraprotein, which is essentially a single antibody excessively produced by abnormal plasma cells.

MM is a highly heterogeneous cancer marked by clonal diversity^[14]^. It starts with underlying germline events, followed by primary - frequently initiating - and secondary genomic aberrations that lead to tumor progression. Using integrated genomics on newly diagnosed patients with MM, Walker *et al*.^[15]^, for example, identified 63 driver genes, including the most diverse oncogenes, such as *FGFR3*, *DIS3*, *PRKD2*, *CCND1*, *IRF4*, *MAF*, *BRAF*, *DIS3*, *ATM*, *FAM46C* and *MYC*. Among the most relevant secondary events, deregulated *MYC* activity is associated with disease progression^[10]^ and occurs in a large percentage (67%) of MM case^[16]^. In one of the largest genome-wide association studies to date, researchers have found several single-nucleotide polymorphisms associated with *MYC* activation, considered a critical exacerbating event and related to poor outcomes^[4]^. Secondary translocations encompassing the *MYC* gene are late progression events that involve an Ig enhancer in 60% of the cases (either the heavy or light chains) and seemingly no other recurrent chromosomal loci in the remaining 40% cases^[17]^.

The heterogeneity of MM imposes a big challenge for its treatment with tailored therapies, which are typically directed against a unique target. Besides, as mentioned above, patients commonly relapse after receiving first-line therapy, often due to a selective pressure exerted by the treatment leading to resistant subclones outgrowth^[18]^. This is the main reason researchers are currently proposing alternating the use of therapies with different mechanisms of action, which could overcome future relapses.

Another critical aspect of MM pathophysiology is its dependence on the BM niche. Probably one of the most critical interactions in the creation of a favorable microenvironment for MM cells proliferation, survival and apoptosis resistance is their relationship with BM stromal cells (BMSCs)^[4]^. The cell-cell crosstalk through adhesion molecules such as VCAM1 and VLA-4 expressed by BMSCs and MM cells, respectively, results in the secretion of cytokines responsible for the formation of an appropriate cancerous milieu. This interaction also accounts for bone destruction, a hallmark of late-stage myeloma that significantly deteriorates the quality of life of MM patients^[19]^. Indeed, the development of osteolytic lesions, present in more than 80% of myeloma patients^[20]^, is one of the most devastating consequences of advanced MM, caused by an imbalance between bone formation and resorption, partly due to a significant reduction in circulating osteoprotegerin^[21]^.

Taken together, all these pieces of evidence appeal for the research of new therapeutic options, either directed against novel targets or focused on bone disease management, to achieve fewer side effects. These new therapies could be combined or sequentially administered together with already approved drugs to render myeloma a preventable or, even better, curable disease.

### Personalized medicine and its limitations

Oncology is one of the most invested fields in discovering new drug options, in an insatiable search for alternative therapies able to overcome the limitations of the already existing ones^[22]^. Personalized medicine, also known as precision medicine, aims to design tailored treatments against major molecular drivers of different pathologies^[23]^. Hence, precision oncology performs molecular profiling of tumors to identify alterations that can be translated into actionable targets^[24]^.

The first step in the profiling is to stratify the patients using new technologies, such as next generation sequencing or multi-omics approaches, to choose the most appropriate treatment for each individual based on their unique molecular aberrations. Very often, the most affected proteins in cancer are kinases, involved in many physiological processes of the cell^[23]^. One well-characterized example is the BCR-ABL gene fusion in chronic myeloid leukemia (CML). This chromosomal defect, known as the Philadelphia chromosome, is the signature of CML, present in all patients suffering from this condition^[25]^. Its discovery led to the development of imatinib, a selective inhibitor of the constitutively active tyrosine kinase resulting from the gene fusion^[26]^. Thanks to imatinib, the survival rates for CML patients notably improved to 90% over 5 years and 88% over 8 years^[25]^. Other tyrosine kinase inhibitors then became very popular because of the broad involvement of the kinome in different malignancies. Thus, numerous small molecules against the enzymatic core or binding pockets of these proteins have been developed ever since, changing the clinical management of cancer^[27]^.

Currently, over 55 targeted therapies are approved to treat different hematologic malignancies^[28]^. As such, they are directed against actionable molecules identified to play essential roles in the biology of immune cells or represent proteins that are highly expressed in these types of tumors. They can be categorized according to the type of target they inhibit: (1) B-cell surface markers [e.g., rituximab, an anti-CD20 monoclonal antibody (mAb), or daratumumab, an anti-CD38 mAb]; (2) survival or proliferation factor receptors (e.g., siltuximab, an anti-IL-6 mAb); (3) cell signaling markers (e.g., ibrutinib, a BTK inhibitor, or idelalisib, a PI3K inhibitor); (4) cell cycle, apoptosis and proteasome machinery (e.g., bortezomib, a proteasome inhibitor, or venetoclax, a Bcl-2 inhibitor); (5) metabolism (e.g., lonidamine, a hexokinase inhibitor); and (6) microenvironment (including immune modulators, such as plerixafor, an anti-CXCR4; pembrolizumab, an anti-PD-1 antibody; CAR-T cells; or bispecific antibodies)^[29]^.

However, as mentioned above, despite the initial success of all these different therapeutic strategies, most patients eventually relapse. Hence, the emergence of drug resistance is not only limited to conventional chemotherapy, but it extends to drugs with a targeted mode of action as well^[30]^. There are several mechanisms of resistance that have been primarily studied and described, including drug efflux, acquired mutations that impair drug binding, trapping in acidic vesicles, enhanced metabolism, activation of compensatory signaling pathways or remnant quiescent stem cells that are inherently resistant^[31]^.

**Figure 2:**
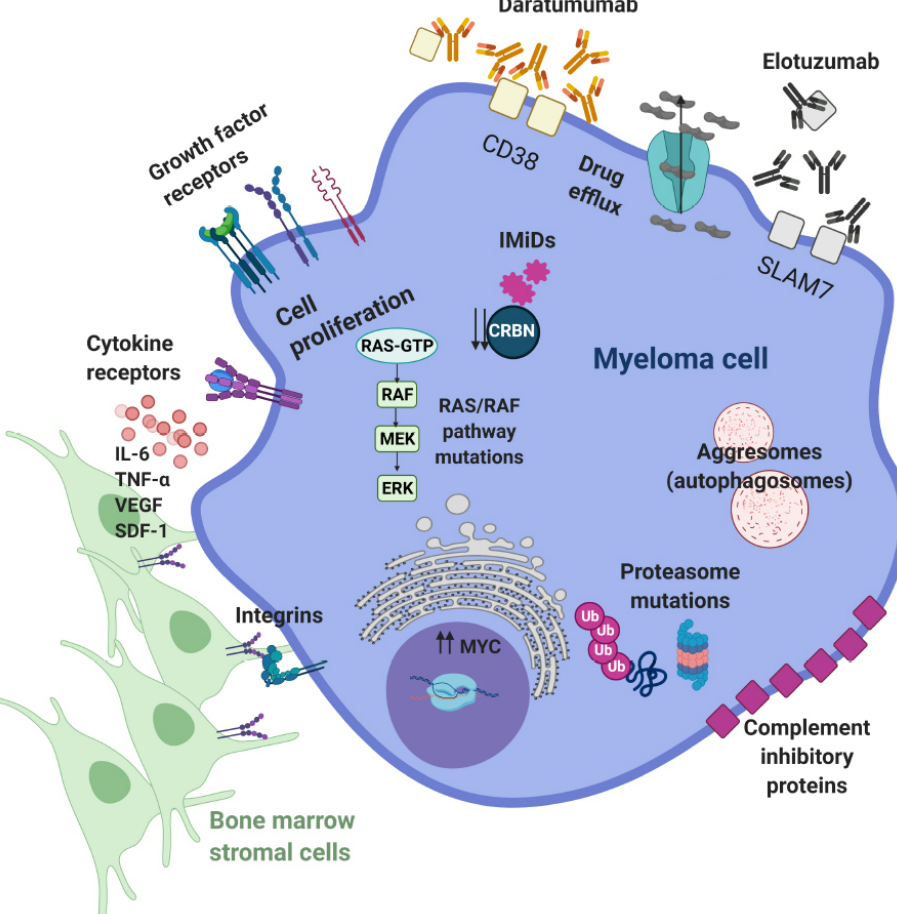
Examples of the most common resistance mechanisms to multiple myeloma therapies. See text for details. For a more thorough description of the resistance mechanisms, check the review from Wallington-Beddoe^[32]^. Figure adapted from^[32]^. CD38: Cluster of differentiation 38; CRBN: cereblon; ERK: extracellular signal-regulated kinases; IMiDs: immunomodulatory agents; IL-6: interleukin-6; MEK: mitogen-activated protein kinase; RAF: rapidly accelerated fibrosarcoma; SDF-1: stromal cell-derived factor; SLAM7: signaling lymphocytic activation molecule family member 7; TNFα: tumor necrosis factor alpha; Ub: ubiquitin; VEGF: vascular endothelial growth factor.

MM, in particular, is an excellent example of frequent disease recurrence through multiple compensatory mechanisms [Figure 2]. For instance, the occurrence of mutations in the proteasome machinery and the RAS/RAF signaling pathway confers resistance to proteasome inhibitors (PIs, e.g., bortezomib) and immunomodulatory agents (IMiDs, e.g., lenalidomide), respectively. Similarly, sequestrations of drugs in autophagosomes or active pumping to the outside are typical resistance mechanisms observed in MM. In addition, MM cells can get protection from the microenvironment by strengthening the interaction with supporting cells through integrins and other adhesion molecules or by increasing proliferation and survival signaling. Lastly, a reduction in the expression levels of certain proteins such as cereblon, CD38 and SLAM7 impairs the activity of targeting agents. Interestingly, CD38 and SLAM7 can also be secreted, acting as decoy receptors for mAbs (daratumumab or elotuzumab), which can be further weakened by increased expression of complement inhibitory proteins that hamper their ability to activate the complement-dependent cytotoxicity^[32]^.

In this scenario of multiple resistance mechanisms, liquid and on-treatment standard biopsies would clearly be beneficial for the identification of biomarkers of resistance or response, to intervene ahead of the point of no return, for instance by switching to a different drug and dosing regimen or even deciding to start combinations with other therapies^[33]^.

### MYC as a key regulator in cancer

*MYC* is one of the most powerful oncogenes found to be deregulated in over half of human cancers^[34]^. The *MYC* gene encodes for a family of basic helix-loop-helix leucine zipper (bHLHZ) transcription factors (TFs), comprising *c-MYC*, *L-MYC* and *N-MYC*, which conduct partially redundant functions depending on the tissue where they are expressed^[35,36]^. To mediate the many biological processes in which it is involved, MYC forms transcriptionally active dimers with its obligate partner MAX, and together they bind DNA at sequences known as E-boxes^[37]^.

MYC displays characteristics of an intrinsically disordered protein in its monomeric form in solution, being mostly unstructured. It comprises an N-terminal transcriptional activation domain, followed by a canonical nuclear localization signal and a C-terminal bHLHZ domain, which is mainly unfolded until it dimerizes with MAX^[37,38]^.

The physiological functions of MYC include, but are not restricted to, cell proliferation and growth, apoptosis, differentiation, migration, stem cell biology, metabolism and transcriptional control over the non-coding transcriptome (miRNAs and lncRNAs)^[39-42]^. Of note, unlike in the case of other TFs and signaling molecules, in which loss of individual proteins can often be compensated by other members of the same pathway or a parallel one, MYC function is non-redundant, as demonstrated by the lethality observed in *MYC* deficient mouse embryos^[43]^.

**Figure 3:**
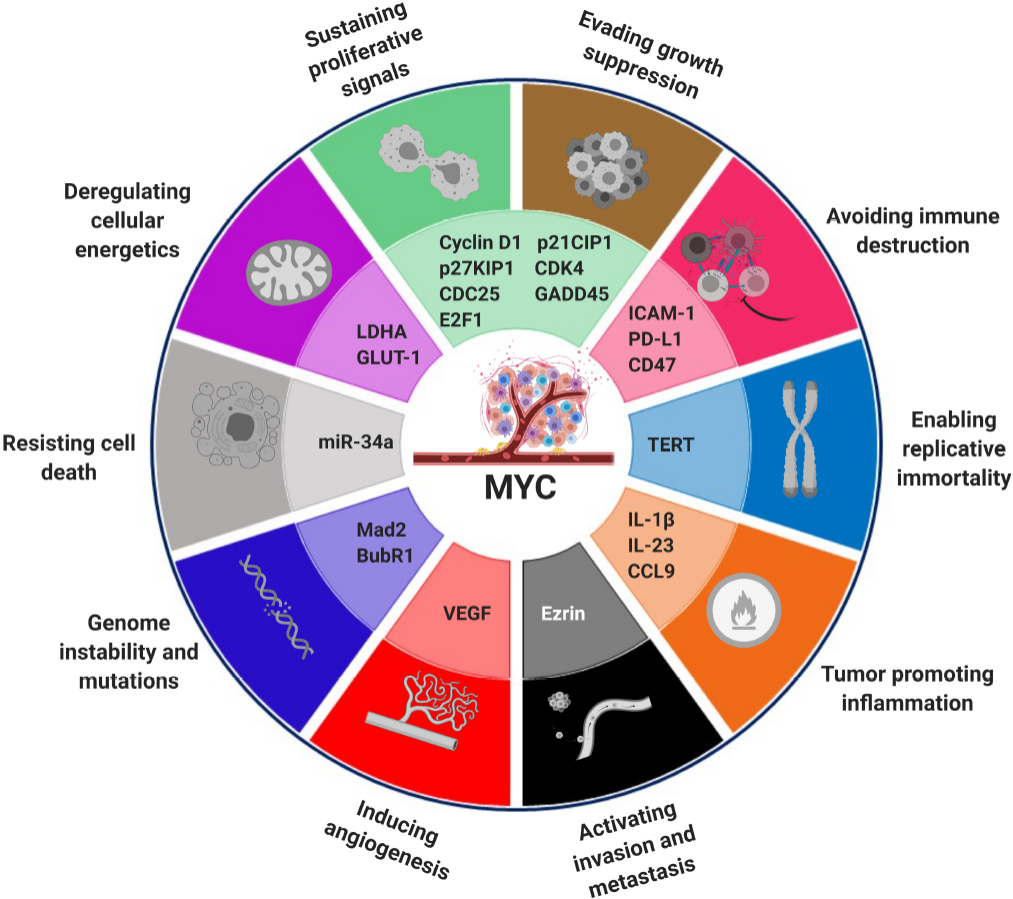
*MYC* as a central node in the hallmarks of cancer. MYC is a transcription factor and master regulator of the expression of around 30% of all human genes. As such, it instructs the differential expression of many genes, contributing to the acquisition of cancer-like properties, as defined by Hanahan and Weinberg^[49]^. In the image, some examples of MYC target genes involved in the tumorigenesis process are indicated next to the hallmark they impinge on. Figure adapted from^[50]^.

Given the highly central role MYC plays in cell proliferation, its expression is tightly regulated at the transcription, mRNA and protein levels^[37]^. Nonetheless, many of the genetic alterations that occur in cancer uncouple *MYC* expression from these usual regulatory constraints: either constitutive activation of signal transduction pathways (e.g., Notch, Wnt and receptor TKs) or direct alterations of *MYC*, such as point mutations, leading to protein stabilization, amplifications or translocations^[37,44]^, can lead to its deregulation. Interestingly, however, deregulation of MYC alone is not always enough to induce tumorigenesis, so that some other genetic alterations are required. The reason is that continuous expression of *MYC* usually has a dual effect, inducing proliferation at first, followed by proliferative arrest, senescence or apoptosis^[45,46]^, so these fail-safe mechanisms need to be disabled in order for MYC to exert its full pro-tumorigenic function. Only then, aberrant expression of its target genes promotes deregulated entry and exit of the cell cycle, increased cell mass through protein biogenesis, restraint of the host immune response, relentless DNA replication, remodeling of the microenvironment, activation of the angiogenic switch, and suppression of the response to autocrine and paracrine regulatory programs and metabolic rewiring [Figure 3]. Hence, *MYC* activation seems to constitute the main inducer of most molecular hallmarks of cancer^[42,45,47,48]^.

In MM, increased MYC activity is related to disease progression^[16,51]^. Such deregulation can happen through many different processes - some of which are mentioned above, such as translocations or sustained activation of upstream signaling pathways, as more extensively reviewed elsewhere (Jovanović *et al*.^[16]^).

Many researchers worldwide have experimentally confirmed MYC’s role in carcinogenesis, in many cases using conditional expression of *MYC* in different tissues with *in vivo* models, including studies on the reversibility of the process upon *MYC* withdrawal^[52-55]^. In these studies, suppression of *MYC* has proven to induce tumor regression not only in those tumors considered *MYC*-driven^[52,56-58]^, but also in those in which *MYC* is not the initiating oncogenic lesion^[59,60]^, suggesting that MYC inhibition would represent an effective treatment for many cancer types.

## MYC INHIBITION STRATEGIES

Extensive evidence in the literature supports the idea that MYC inhibitors would have a huge impact on cancer treatment. However, no such drug is available in the clinic yet. As it happens, MYC is still considered “undruggable”, a term coined for proteins believed not pharmacologically targetable^[61]^.

The reasons are multiple:

(1) The MYC family comprises three potentially redundant members so that complete MYC inhibition would require simultaneous blockade of all three at a time.

(2) MYC is a mainly unstructured TF, lacking a binding pocket to tamper with, and it functions mainly through protein-protein interactions (PPIs), so that targeting it with the classical small molecule therapeutic design is hardly achievable, in part due to their small interacting surface.

(3) Its localization in the nucleus supposes another challenge since the inhibitory compound would need to reach this subcellular compartment to exert its function.

(4) Finally, given the pivotal role of MYC not only in tumor biology but also in physiological conditions, it was thought that MYC inhibition could potentially cause catastrophic adverse effects in healthy tissues^[62]^.

However, thanks to the design of Omomyc, a dominant-negative mutant of the bHLHZ of *MYC* and the best direct MYC inhibitor known to date^[63,64]^, we managed to prove most of these assumptions wrong. Indeed, Omomyc has been extensively characterized and validated in various cancer models, both as a transgene and as a mini-protein (for a historical perspective, refer to^[65]^) and demonstrated the feasibility, safety and dramatic therapeutic impact of MYC inhibition.

**Figure 4:**
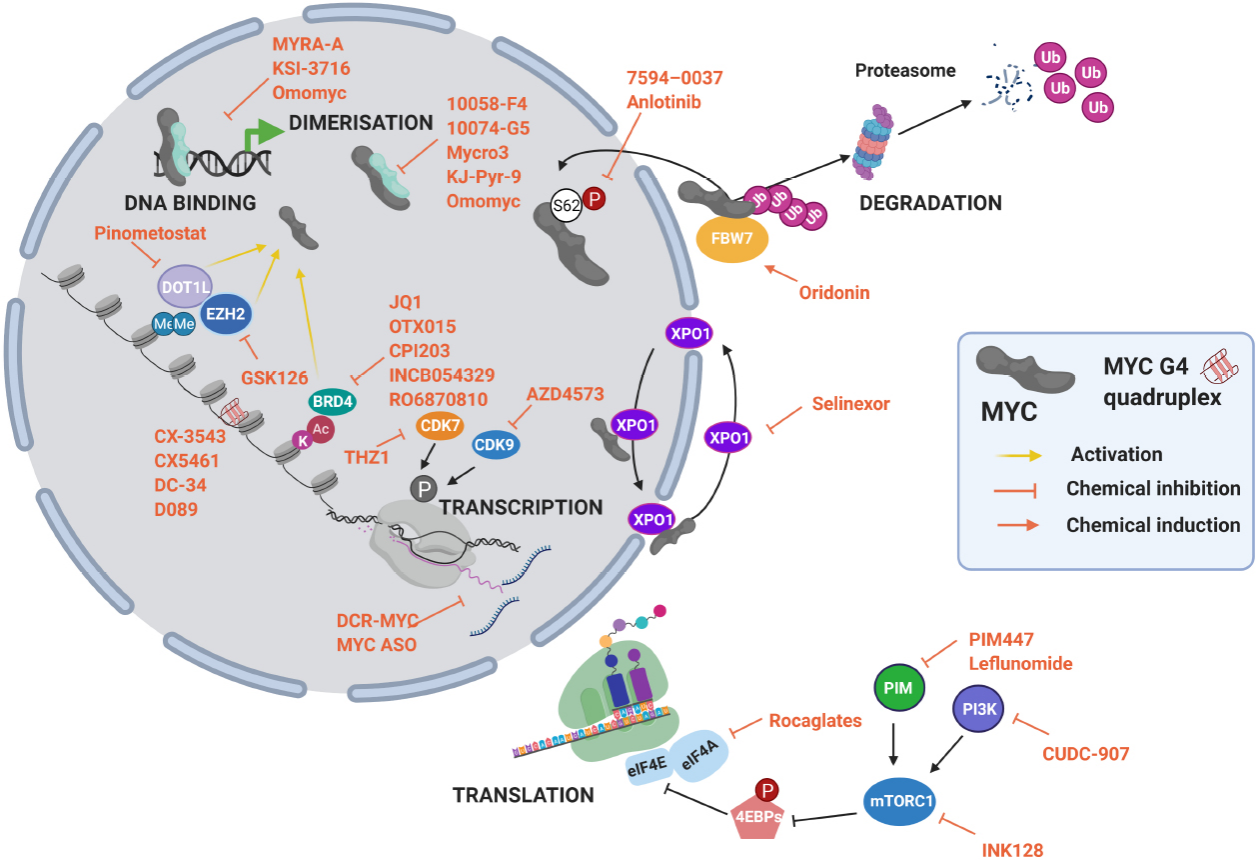
MYC inhibition strategies at different levels of MYC life cycle. Some examples of drugs are listed. Figure adapted from^[66]^.

However, Omomyc is not alone, and several investigations were or are being conducted to develop other drug candidates against this TF. The strategies used can be classified as indirect or direct MYC inhibitors, as summarized in Figure 4. In Table 1 we have synthesized the preclinical and clinical studies that encompass the specific indirect MYC inhibitors detailed in the text. In this review, we describe several inhibitors used in the context of MM, independently of their stage of development, aiming at summarizing the lesson learned from each of them.

**Table 1:** Summary of the different indirect MYC inhibitors and their development stage for hematological tumors (focused on MM)

**Class of inhibitor**	**Name**	**Combined with other therapies?**	**Mechanism of action**	**Clinical trials**	**Ref.**
BET inhibitors	OTX015	PIs, IMiDs and chemo	Downregulation of BRD4 Interruption of pathways and genes critical for MM survival and resistance (e.g., NF-κB, c-MYC)	Preclinical testing	Gu *et al*.^[67]^
	CPI203	IMiDs	Downregulation of MYC, Ikaros and IRF4	Preclinical testing	Díaz *et al*.^[68]^
	AZD7543 and ARV825	Combination of CDK9 inhibitor and BET PROTAC	Downregulation of BRD2, BRD4, MYC and phosphorylated RNA polymerase II	Preclinical testing	Lim *et al*.^[69]^
	INCB054329	JAK inhibitors^†^	Reduced expression of IL6R and STAT3 signaling. Downregulation of c-MYC*, *FGFR3 and NSD2/MMSET/WHSC1^†^	Discontinued (NCT02431260)^‡^	^†^Stubbs *et al*.^[70]^ ^‡^Falchook *et al*.^[71]^
	RO6870810	No	High affinity for the acetyl-lysine recognition pocket of BET family (BRD4, BRD3, BRD2 and BRDT)	Being evaluated (NCT03068351)	Shapiro *et al*.^[72]^
Epigenetic modulators (continued)	GSK126	No	Increased IFN signaling and stopped IRF4-MYC axis^†^	Terminated (the maximal dose and schedule attained with GSK2816126 showed insufficient evidence of clinical activity and did not justify further clinical investigation) (NCT02082977)^‡^	^†^Ishiguro *et al*.^[73]^ ^‡^Yap *et al*.^[74]^
	SGC0946	No, but the authors suggest that KO of SETD1B increases the sensitivity to DOT1L inhibition	Suppression of IRF4-MYC, ATF4, global protein synthesis and alteration of ER stress pathways	Preclinical	Dafflon *et al*.^[75]^
	Panobinostat	PIs and IMiDs^‡^	Downregulation of HO-1, IRF4 and MYC^†^	Active (the results highlight the ability of resensitizing patients with acquired resistance) (NCT01965353)^‡^	^†^Tang *et al*.^[76]^ ^‡^Laubach *et al*.^[77]^
	IRF4 ASO	No	Decrease of MYC and MYC targets	Recruiting (NCT04398485)	Mondala *et al*.^[78]^
	RRX-001	Bortezomib, pomalidomide, HDAC inhibitor SAHA	Causative of oxidative stress in hypoxia, inhibiting global hypermethylation	Preclinical	Das* et al*.^[79]^ Cabrales *et al*.^[80]^
CDK7/CDK9 inhibitors	THZ1	PIs and BH3-mimetics	Downregulation of c-MYC, MCL-1 and BCL.X_L_	Preclinical	Zhang *et al*.^[81]^
	CDK9i (cpds 66 and 68)	No	CDK9 inhibition in the low nanomolar range	Preclinical	Czudor *et al*.^[82]^
	SY-1365	Venetoclax (BCL2 inhibitor)	Lowering of the MCL-1 protein and alteration of cell cycle and DNA repair pathways	Solid tumors: terminated (business decision) (NCT03134638)	Hu *et al*.^[83]^
	AZD4573	Venetoclax	Depletion of MCL-1	Recruiting (NCT03263637)	Cidado *et al*.^[84]^
mTOR/PI3K inhibitors	CUDC-907	Dual HDAC and PI3K inhibitor	Decrease of MYC protein levels	Completed (results information submitted but is not yet publicly available on ClinicalTrials.gov.) (NCT02674750)	Sun *et al*.^[85]^
	PIM-447	IMiDs and PIs^†^	Decreased levels of MYC and increased MAD-1. Disruption of eIF4e and downregulation of IRF4^†^	Completed (promising single-agent activity and potential to combine with other agents) (NCT01456689)^‡^	^†^Paíno *et al*.^[86,87]^ ^‡^Raab *et al*.^[88]^
	Leflunomide	Lenalidomide^†^	Downregulation of MYC and inhibition of TKs^†^	Active (stable disease in 9/11 patients) (NCT02509052)^‡^	^†^Buettner *et al*.^[89]^ ^‡^Rosenzweig *et al*.^[90]^
eIFs/nuclear export inhibitors	Selinexor	Low dose of dexamethasone	Inhibition of XPO1 = retention of tumor suppressors and reduction of oncoproteins translation	Completed (objective treatment responses) (NCT02336815)^a^ Active (improved PFS and ORR and reduced peripheral neuropathy) (NCT03110562)^b^ Recruiting (NCT04414475)	^a^Vogl *et al*.^[91]^ ^a^Chari *et al*.^[92]^ ^b^Mateos *et al*.^[93]^ ^b^Richard *et al*.^[94]^ ^b^Auner *et al*.^[95]^ ^b^Grosicki *et al*.^[96]^
	Rocaglates	ABT-199 and dexamethasone	Blockage of *MYC* mRNA translation	Preclinical	Maïga *et al*.^[97]^
Promoters of MYC degradation (continued)	TAZ	No	MYC loss	Preclinical	Grieve *et al*.^[98]^
	NSC12	Bortezomib	Mitochondrial oxidative stress and DNA damage	Preclinical	Ronca *et al*.^[99]^
	Erdafitinib	PI and IMiDs	Selective TK FGFR inhibitor	Recruiting (NCT03732703)	Ronca *et al*.^[99]^
	AZD4547	No	Selective TK FGFR inhibitor	Completed (limited activity and low ORR) (NCT04439240)	Chae *et al*.^[100]^
	7594-0037	No	Reduction of MYC phosphorylation on serine 62 and of its stability	Preclinical	Yao *et al*.^[101]^
	Anlotinib (AL3818)	No	Reduction of MYC phosphorylation on serine 62 and phosphorylation of threonine 58	Preclinical Clinical stage for other indications	Cao *et al*.^[102]^ Shen *et al*.^[103]^
	Rapamycin + MS-275	mTORi + HDACi	Decreased MYC stability	Preclinical	Simmons *et al*.^[104]^

^†^Refers to preclinical data. ^‡^Indicates clinical data. ^a^Refers to NCT02336815. ^b^Refers to NCT03110562. BET: Bromodomain and extra-terminal; PIs: proteasome inhibitors; IMiDs: immunomodulatory drugs; NF-κB: nuclear factor kappa-light-chain-enhancer of activated B cells; IRF4: interferon regulatory factor 4; CDK9: cyclin-dependent kinase 9; PROTAC: proteolysis targeting chimera; IL6R: interleukin-6 receptor; FGFR3: fibroblast growth factor receptor 3; IFN: interferon; KO: knock out; ATF4: activating transcription factor 4; ER: endoplasmic reticulum; TK: tyrosine kinase; mTORi: mTOR inhibitors; HDACi: histone deacetylase inhibitors; ASO: antisense oligonucleotides; eIF: Eukaryotic translation Initiation Factor; PFS: progression free survival; ORR: overall response rate; HO-1: heme oxygenase-1; MM: multiple myeloma.

### Indirect MYC inhibitors

Because the MYC protein itself appeared to be a “slippery as an eel” target, researchers mostly opted for indirect pharmacological approaches, targeting its transcription, translation or degradation.

### Blockade of MYC transcription

#### Bromodomain and extra-terminal motif inhibitors

Bromodomain and extra-terminal (BET) inhibitors (BETis) were found to alter the transcription of the *MYC* gene, which is regulated by BET proteins such as BRD4. The first compound to show this effect was JQ1, a BETi widely used in *in vitro* studies. JQ1 significantly downregulated *MYC* expression, causing a reduction in tumor burden and extending the overall survival in a MM mouse model^[105]^. Several second-generation BETis with improved properties (e.g., superior oral or intraperitoneal bioavailability, distinct chemical structure, *etc*.) have been tested in preclinical models of MM either as monotherapy or combined with other agents.

OTX015 was recently demonstrated to have a considerable antitumor effect in a myeloma xenograft model that recapitulates the disseminated form of the human disease, extending the overall survival of mice^[106]^. The authors found that MYC levels were downregulated in two myeloma cell lines, which suggested that MYC itself could be a pharmacodynamic (PD) marker of BET inhibition, although its usefulness would be limited to hematologic tumors^[107]^. In a more recent study, Gu *et al*.^[67]^ combined OTX015 with different antimyeloma agents (PIs, IMiDs or chemotherapy), showing that attacking the biology of myeloma cells through distinct angles could hold promise to render MM cells sensitive.

Similarly, Díaz *et al*.^[68]^ combined another BETi, CPI203, with lenalidomide and dexamethasone, two IMiDs usually used as myeloma standard therapy. This combination led to complete tumor growth arrest, accompanied by a significant reduction in MYC, IRF4 (interferon regulatory factor 4) and Ikaros positive cells by immunohistochemistry.

Lim *et al*.^[69] ^were the first to combine the mechanisms of action of a CDK9 inhibitor (AZD 4573) and a BET PROTAC (ARV 825), showing synergy between them in MM. They demonstrated a blockage in proliferation and enhanced apoptosis by decreasing the phosphorylation of RNA Polymerase II, reduction of MCL-1 and MYC proteins and MYC downregulation, resulting in a very significant tumor burden reduction.

In 2019, Stubbs *et al*.^[70]^ tested INCB054329 in various myeloma cells, alone or combined with JAK inhibitors, showing better efficacy than either compound alone in *in vivo* models, suggesting that the intelligent design of novel combination approaches could overcome the tumor heterogeneity and complexity. Interestingly, Falchook *et al*.^[71]^ reported that INCB054329, when evaluated as monotherapy in a Phase I/II study (NCT02431260), required a second round of titration due to the high interpatient variability observed in clearance and exposure at different doses. However, the starting 20 mg twice a day dose was not tolerated, and, unfortunately, several patients (MM participant included) discontinued treatment.

One other inhibitor, RO6870810, has been studied in a Phase I trial (NCT03068351). In the study, patients have reached pharmacokinetic (PK) exposures related to PD effects, displaying thrombocytopenia - a common side effect of BETi - but the final results are still being evaluated^[72]^.

As mentioned above, unfortunately, the success of BETis in the clinical setting has been limited so far, in part due to dose-limiting toxic effects. It should be noted that BETis also target key transcriptional networks controlled by tissue- and disease-specific enhancer regions, so that the transcriptional effects of BET inhibition are highly context-dependent^[108]^. Although many molecules of this class are being evaluated in Phase I/II clinical trials (e.g., CPI-0610 and RO6870810), they are clearly not considered as *MYC* inhibitors only. For a deeper analysis of the different studies in humans, as well as their NCT identifiers, refer to the following reviews^[109-112]^.

#### Epigenetic inhibitors

Chromatin modifiers such as histone methyltransferases have been defined as promising targets for many cancer types, including hematologic malignancies^[113]^, although not necessarily through MYC inhibition only. MYC, in particular, has been described to interact with chromatin complexes and to be able to induce epigenetic modifications. For instance, Tu *et al.*^[114]^ recently defined the G9a methyltransferase as one of MYC interactors. Similarly, EZH2 and DOT1L are other histone methyltransferases whose inhibition leads to MYC downregulation, resulting in tumor growth suppression through slightly different mechanisms^[115,116]^.

In particular, EZH2 is involved in myeloma’s pathogenesis and the acquisition of resistance to different drugs, and it is related to poor prognosis, raising interest in inhibiting it. Strong preclinical data support the translatability of this approach to a clinical setting^[115]^, including a recent report in which Ishiguro *et al.*^[117]^ showed that the dual EZH2/G9a inhibitor GSK126 exerted a potent tumor-suppressive effect by activating an immune response through upregulation of interferon (IFN) signaling and by halting the IRF4-MYC axis. In a separate earlier publication, the same authors showed that DOT1L inhibitors function in a very similar fashion, causing DNA damage that could explain the stimulation of IFN signaling, while shutting down the IRF4-MYC axis and thus blocking MM growth^[73]^. Interestingly, Dafflon *et al*.^[75]^ characterized the mechanism of action of SGC0946 and identified a subset of MM cell lines susceptible to it. In addition to the suppression of the IRF4-MYC axis, sensitive cells showed suppression of the TF ATF4, reduced global protein synthesis and alterations in the ER stress pathway^[75]^. In fact, panobinostat, a broad-spectrum histone deacetylase (HDAC) inhibitor, functions with almost the same mechanism of action, downregulating the mRNA of heme oxygenase-1, as well as IRF4 and MYC, causing the arrest of primary CD138^+^ patient cells and inducing their apoptosis^[76]^. Indeed, panobinostat is one of the few epigenetic drugs approved by the FDA for the treatment of MM^[113]^. Incidentally, Mondala *et al.*^[78]^ recently targeted IRF4 in MM as well, using instead an antisense oligonucleotide (ASO) and causing a decrease in MYC and MYC targets. Importantly, Mondala *et al.*^[78]^ reported eradication of myeloma progenitors and malignant plasma cells and abrogation of tumor formation and disease dissemination in xenograft models while displaying no effect on normal human hematopoietic stem cells.

A very different type of molecule, RRx-001, was defined by its creators as a MYC inhibitor^[118,119]^ and “erythrophagoimmunotherapeutic”. RRx-001 is also a novel hypoxia-selective epigenetic agent derived from the aerospace industry^[120]^. Its mechanism of action differs from that of the hypomethylating agents azacitidine or decitabine, in that RRx-001 causes oxidative stress under hypoxic conditions, inhibiting global hypermethylation and restoring tumor suppressor gene function^[79]^. It has been shown to exert antitumor activity in a myeloma xenograft model, both as monotherapy or in combination with standard antimyeloma agents (glucocorticoids or PIs)^[79,80]^. The authors describing it clarified that its antineoplastic efficacy is explained by a variety of mechanisms that include promoting an immune response, epigenetic modifications, apoptosis, antioxidant and antiangiogenic activities and acting as a nitric oxide donor^[120]^. Currently, this molecule seems to be mostly employed against solid tumors rather than hematological diseases.

Unfortunately, as standalone strategies, none of the above molecules performed in the clinical setting as predicted by preclinical studies. In a Phase I clinical trial, the investigators concluded that GSK126 is not a suitable EZH2 inhibitor to treat advanced solid or hematologic malignancies due to its improvable PK profile, leading to inefficient exposure at tolerated doses and displaying a minimal anticancer activity^[74]^. Similarly, Stein *et al.*^[121]^ evaluated the safety and efficacy of pinometostat in another Phase I study of acute mixed lineage leukemia and observed that, despite reaching exposure to anticancer levels, it could only display minor clinical activity, suggesting that its combination with standard of care agents would be a better choice. In support of this hypothesis, a recent Phase I study for the combination of panobinostat with several myeloma standard therapies demonstrated the ability of the combination to resensitize relapsed/refractory MM patients, in the context of resistance to IMiDs, PIs or other novel targets^[77]^. In addition, the IRF4 ASO will soon be tested in a first-in-human clinical trial as monotherapy for relapsed/refractory myeloma to assess its safety, tolerability and antimyeloma activity (NCT04398485).

#### Cyclin-dependent kinase 7or 9 inhibitors

The blockade of cyclin-dependent kinase 7or 9 (CDK7 or CDK9) can also downregulate MYC expression and reduce mRNA levels by inhibiting RNA polymerase II-dependent transcription affecting the stability of preinitiation complexes^[122]^. Some studies demonstrated that THZ1, a covalent CDK7 inhibitor, can indeed suppress master transcription-regulating oncogenes, such as MYC, in neuroblastoma models^[123,124]^, and Zhang *et al.*^[81]^ expanded the study to several myeloma cell lines, reporting a potent antiproliferative and proapoptotic effect with exposure times as little as 24 h and even in the context of resistance to bortezomib. Mechanistically speaking, apart from MYC and DNA damage response genes (CtIP, FANCD2, RAD51, BRAC1 and ERCC1), the inhibitor diminished the expression of MCL-1 and BCL-X_L_, in line with its expected function as a CDK7 and CDK12/13 inhibitor^[81]^. Notably, the authors demonstrated a reduction of subcutaneous xenograft tumors and extended survival of mice upon treatment with 10 mg/kg of THZ1 and suggested the combination with PIs or B-cell lymphoma 2 (Bcl-2) Homology 3 (BH-3) mimetics, as they observed a significant increase in cell death of primary patient-derived CD138^+^ cells and MM stem-like cells (CD138^-^, CD19^+^, CD20^+^, CD27^+^), while having little effect on healthy cord blood cells (CD138^-^, CD34^+^)^[81]^. In a brief article, Czudor *et al*.^[82]^ synthesized two novel CDK9 inhibitors that also displayed considerable antiproliferative effects in MM cell lines.

Thanks to the antitumor effect shown by these molecules at the preclinical stage, the first covalent CDK7 inhibitor (SY-1365) entered clinical trials in 2017 for the treatment of solid tumors^[83]^. Unfortunately, the study was terminated in March 2021 based on a business decision (NCT03134638). On a more encouraging note, however, the CDK9 inhibitor AZD4573 has been evaluated against a panel of hematologic cancer models, including cancer cell lines and cell line- and patient-derived xenografts, both subcutaneous and disseminated models. It demonstrated a similar mechanism of action to that shown for THZ1, depleting MCL-1^[84]^. The definition of the PK/PD/efficacy model helped inform the currently ongoing clinical trial design to assess the safety, tolerability, PK and preliminary anticancer activity of this molecule against relapsed/refractory hematologic malignancies (NCT03263637).

In any case, how much of the therapeutic impact of the above-mentioned drugs is due specifically to MYC inhibition remains to be established.

### Blockade of MYC mRNA translation

#### Mammalian target of rapamycin and PI3K inhibitors

Mammalian target of rapamycin (mTOR) seems to play an essential role in the translation of MYC mRNA^[125]^, among others, laying the foundations for inhibiting the PI3K/mTOR pathway as another possible strategy to indirectly inhibit MYC. Indeed, several drugs have already been approved (e.g., everolimus, temsirolimus and torkinib), and some others are in earlier clinical developmental stages (e.g., INK128)^[126]^. Interestingly, the dual PI3K/HDAC inhibitor CUDC-907 showed potent suppressive activity against MYC-dependent tumors^[85,127]^, and, in a Phase II study assessing its safety (NCT02674750), 14% of evaluable relapsed/refractory lymphoma patients achieved an objective clinical response^[85]^.

In this same context, Paíno *et al.*^[86]^ presented a pan-PIM kinase inhibitor, PIM447, able to block all three PIM kinases and act as an mTOR inhibitor, which exhibited cytotoxic effects on myeloma cells and a bone-protective effect in a disseminated mouse model of human myeloma. Interestingly, the authors also reported the decrease of MYC levels and the increased expression of MAD-1, a MYC antagonist. In line with what others proposed for different small-molecule inhibitors, the authors demonstrated a strong synergistic effect with standard of care agents (IMiDs and PIs), supporting the use of this combination to treat MM patients^[86]^. In fact, in a more recent publication, the same authors used one of these combinations (PIM447 plus pomalidomide and dexamethasone) and showed improved survival in a preclinical mouse model, where they revealed a convergent blockage of MYC and mTORC1, disrupting the function of eIF4e (Eukaryotic translation Initiation Factor 4E), an essential element of the Initiation Translation Complex^[128]^, and downregulating IRF4, important in many immune-related contexts^[129]^, as happened with several BETi^[87]^. Interestingly, Buettner *et al.*^[89] ^presented a similar effect by leflunomide, an orally available, non-toxic, inexpensive immunosuppressive drug regularly used to treat rheumatoid arthritis. Leflunomide downregulated MYC by inhibiting several tyrosine kinases, including the PIM family, and synergized in combination with lenalidomide, another IMiD used as standard therapy in MM, further decreasing MYC and causing MM growth inhibition both *in vitro* and *in vivo*^[89]^.

The first-in-human trial to determine the maximum-tolerated dose (MTD) or recommended dose, safety, PK and preliminary antimyeloma activity of PIM447 in relapsed/refractory myeloma showed that the compound was tolerated by heavily pretreated patients and clinically benefitted 25.3% of the study population (even though most of the discontinuations were due to disease progression)^[88]^. Of note, the investigators could not evaluate the PD markers identified in preclinical studies (phospho-Bad, phospho4EBP1 and c-MYC) because of the absence of clinically valid assays^[88]^. Encouragingly, the progression-free survival seen as monotherapy, together with the preclinical evidence supporting the combination of several agents, builds the case for combining PIM447 with other targeted therapies to overcome drug resistance in patients^[88]^.

Of note, a Phase I clinical trial was also designed to repurpose the use of leflunomide in relapsed/refractory myeloma patients (NCT02509052)^[90]^. As stated by Rosenzweig *et al.*^[90]^, given the stable disease achieved in 9/11 subjects, coupled with the tolerable safety profile, leflunomide could be another option for combinatorial regimens to treat MM or, if introduced at earlier developmental stages (SMM) of the disease, delay its progression into the fully malignant one.

#### Blocking eukaryotic translation initiation factors or nuclear export of mRNA

Selinexor is an oral selective exportin 1 (XPO1) inhibitor that causes tumor suppression of several cancer models through different mechanisms. One of them is the impairment of the nuclear export of tumor suppressor and growth regulatory proteins, such as p53 or MYC, respectively^[130]^. In addition, it has been shown to reduce the cap-dependent translation of several oncogenes, including MYC^[130]^.

Vogl *et al*.^[91]^ tested selinexor in combination with low-dose dexamethasone in relapsed or refractory myeloma patients. In this Phase II trial, patients were either quad-refractory (bortezomib, carfilzomib, pomalidomide and lenalidomide refractory disease) or penta-refractory (anti-CD38 refractory disease)^[91]^. The overall response rate (the primary endpoint) was 21%, very similar in the quad- and penta-refractory patients’ cohorts, providing the latter with a new treatment option. Following the same trend, the authors suggested using selinexor in combination with other standard antimyeloma agents, with the objective of overcoming or at least reducing the appearance of drug resistance^[91]^. Indeed, a couple of randomized Phase II/III trials sponsored by Karyopharm Therapeutics Inc. are currently evaluating the efficacy of the abovementioned combinations (NCT03110562 and NCT04414475).

Rocaglates are secondary metabolites of the plant genus *Aglaia*. These compounds inhibit eIF4A, impeding translation initiation. Maïga *et al*.^[97]^ studied the potency of the synthetic derivative oxo-aglaiastatin (CMLD011580) to inhibit translation and synergize with other compounds against hematologic tumors. CMLD011580 induced cell death of JJN3 and MMS1, two myeloma cell lines, upon combined treatment with ABT-199, a BCL-2 inhibitor. Importantly, the authors showed how the agaliastatin analog could block MYC mRNA translation and synergize with the glucocorticoid dexamethasone (as previously shown by the same group with another rocaglate, silvestrol), inducing cytotoxic effects in MM cell lines. In addition, they explored the effect on primary patient-derived cells, demonstrating a considerable reduction in CD138^+^ cells^[97]^. Despite their apparent applicability as anticancer agents, no current clinical trials are dedicated to studying this therapeutic approach. However, they are being investigated as broad-spectrum antivirals, thanks to their unique mechanism of action and minimal potential toxic side effects while inducing efficient inhibition of RNA viruses^[131]^.

Once again, since all the above-mentioned drugs affect translation of many more proteins besides MYC, ascribing their therapeutic effect to MYC only would be incorrect.

### Promoting MYC degradation

MYC is ubiquitinated by ubiquitin ligases, such as FBW7, which induce its degradation through the proteasome machinery. Inhibition of deubiquitinases that stabilize MYC (e.g., USP28 and USP36)^[132]^ or triggering the FBW7-mediated proteasomal degradation of MYC with oridonin^[133]^ are both potential strategies to indirectly inhibit MYC.

In this context, Grieve *et al.*^[98]^ discovered an unexpected role of the TAZ, a transcriptional coactivator, component of the Hippo-signaling pathway, described to function as an oncogene in many solid cancers, but found to act as a tumor suppressor in myeloma: TAZ expression inversely correlated with the prognostic outcome in myeloma patients. Indeed, Grieve *et al.*^[98]^ demonstrated that restoring TAZ using lentiviruses *in vitro* or pharmacologically upregulating it by treating cells with a hypomethylating drug (DAC) resulted in MM cell death. They also pinpointed the underlying molecular mechanism in the downregulation of MYC at different levels (transcriptomic, proteomic and posttranslational), resulting in the observed antiproliferative effect. Although the exact mechanisms for MYC loss remain elusive, understanding the TAZ-mediated regulation of MYC, especially posttranslationally, could produce valuable information on potential combinations involving the upregulation of TAZ with anti-MYC therapies^[98]^.

Another means to cause MM cell death and overcome bortezomib resistance was described by Ronca *et al*.^[99]^, showing that FGF/FGFR blockade by the pan-FGF trap molecule NSC12 induces mitochondrial oxidative stress and DNA damage. In particular, mitochondrial oxidative stress occurred as a consequence of proteasomal degradation of the c-MYC oncoprotein, which caused glutathione depletion. The authors reported two clinical trials assessing selective TK FGFR inhibitors, erdafitinib and AZD4547, one ongoing and one just completed (NCT03732703 and NCT04439240). It would be interesting to know their effect on tumor MYC levels^[99]^.

Yao *et al*.^[101]^ found 7594-0037, which also led to cell death of MM cells, in a screen of the ChemiDev database in search of MYC inhibitors and showed that 7594-0037 induced MYC degradation by reducing its phosphorylation on serine 62. In addition, the molecule further decreased the MYC intrinsic lack of stability by binding to the N-terminus, preventing the C- and N-termini from interacting^[101]^. The authors showed how treatment with 7594-0037 induced cell cycle arrest in G_2_/M in RPMI-8226 and U266 cells, promoting apoptotic death, as seen by an increase in PARP1, caspase-8 and caspase-9 cleavage. The compound demonstrated also synergistic effects with bortezomib, supporting its use to overcome resistance. Yao *et al*.^[101]^ suggested that this approach has the potential to be translated into the clinical setting. However, extensive research is still required, starting with evaluating the therapeutic impact in animal models, as well as the bioavailability and PK of the compound, to decide whether it is worth pursuing further.

A completely different drug, yet with a similar mechanism of action, is anlotinib (AL3818), a multi-targeting tyrosine kinase inhibitor directed against vascular endothelial growth factor receptor (VEGFR) 1-3, c-Kit, platelet-derived growth factor receptor (PDGFR)-α/β and fibroblast growth factor receptor (FGFR)^[102]^. Cao *et al.*^[102]^ showed the accumulation of RPMI-8226, NCI-H929 and primary patient-derived cells in G_2_/M phase upon treatment with anlotinib, reporting apoptotic cell death seen by cleavage of PARP1 and caspases 3 and 9. Notably, the authors reported the direct interaction of anlotinib with the oncoprotein MYC, leading to its ubiquitin proteasome-mediated degradation, which is triggered by dephosphorylation of serine 62 and phosphorylation of threonine 58. Efficacy was also observed in an *in vivo* subcutaneous xenograft model, in which anlotinib significantly impaired tumor growth, reducing the proliferative marker Ki67 and increasing TUNEL and caspase-3-positive cells^[102]^. This interaction is a new aspect of the inhibitor so far unexplored that sets the rationale for using this inhibitor to treat MYC-deregulated cancers. Hitherto, no clinical trial is approved to evaluate anlotinib against hematologic cancers, although it is being studied for several other indications^[103]^.

Finally, another reported strategy to induce MYC degradation makes use of mTOR inhibition (as already mentioned in this manuscript as a potential way of preventing MYC translation) in combination with HDAC inhibition. Indeed, Simmons *et al*.^[104]^ made us of mTORi (rapamycin) and HDACi (MS-275/entinostat) inhibitors in myeloma, reducing MYC protein stability and causing a significant anti-tumor effect. Simmons *et al*.^[104] ^suggested that the combined use of drug classes that have separately already entered clinical practice as single agents could represent a promising strategy to inhibit MYC.

### Direct MYC inhibitors

As mentioned above, the MYC protein is hard to target with traditional small molecule drugs due to its large, disordered protein interface and lack of deep pockets. In addition, it is often inaccessible to large biologics, which are not always capable of crossing cell membranes.

### Direct inhibition of MYC expression

#### G-quadruplex stabilization

G-quadruplexes are four-stranded DNA structures formed in guanine-rich regions. The *MYC* promoter happens to have such a structure, which acts as a silencer element, repressing its transcription^[134]^. Several studies have shown that some small-molecule ligands (e.g., cationic porphyrins and quindolines) can stabilize such G-quadruplex. The first one was CX-3543, which was shown to reduce *MYC* transcription^[135,136]^. Subsequently, a more selective RNA polymerase I inhibitor, termed CX-5461, demonstrated the ability to be used *in vivo*^[137]^. This compound was even evaluated in a Phase I study for advanced hematologic malignancies and was well tolerated. The predominant population of patients corresponded to myeloma, whose best response was stable disease in half of the cases^[138]^. More recently, Leung *et al*.^[139]^ proposed a novel liposomal formulation of CX-5461 that could result in exposure to higher concentrations of the drug over time, potentially increasing its efficacy.

Others have identified different small molecules that act as MYC G4-stabilizers, such as DC-34^[140]^, D089^[141]^ or DM039^[142]^. Calabrese *et al*.^[140]^ explored the effects of DC-34 in MM cell lines, observing that the compound did not downregulate other G4-dependent genes to the same extent as *MYC*, which was efficiently silenced, with consequent antiproliferative activity. On their end, Gaikwad *et al*.^[141]^ described a cytotoxic effect of D089 in myeloma cells regardless of their protective microenvironment when cocultured with bone marrow stromal cells, and such death occurred through the activation of the ER stress pathway leading to pyroptosis and senescence^[141]^.

#### Antisense oligonucleotides and small interfering RNA

A different strategy to inhibit MYC’s expression is to promote the degradation of its mRNA, thus preventing its translation. For this purpose, investigators have used antisense oligonucleotides (INX-3280)^[143]^ or antisense oligomers (AVI-4126)^[144]^.

With a similar mode of inhibition, the lentiviral delivery of shRNA^[145]^ or RNAi encapsulated in a lipid nanoparticle (DCR-MYC)^[146]^ can also result in the elimination of *MYC *mRNA. Even though DCR-MYC reached clinical trials, the efficacy results obtained did not fulfill the company’s expectations, thus its development was ultimately discontinued (NCT02110563).

In Table 2, we have summarized the most recent studies, mostly preclinical, about direct MYC inhibitors in hematologic malignancies, particularly MM.

**Table 2:** Summary of the different direct MYC inhibitors and their development stage for hematological tumors (focused on MM)

**Class of inhibitor**	**Name**	**Combined with other therapies?**	**Mechanism of action**	**Clinical trials**	**Ref.**
G4-quadruplex stabilizers	CX-5461	No	Reduction of *MYC* transcription	Beneficial clinical responses in some cases and MTD determination (ACTRN12613001061729)	Khot *et al*.^[138]^
	DC-34	No		Preclinical	Calabrese *et al*.^[140]^
	D089	No		Preclinical	Gaikwad *et al*.^[141]^
	DMO039	No		Preclinical	Minard *et al*.^[142]^
siRNA	DCR-MYC	No	Elimination of *MYC* mRNA	Terminated (sponsor decision) (NCT02110563)	Tolcher *et al*.^[146]^
PPI or DNA binding inhibitors		No	Blockade of the interaction of MYC with partners (mainly MAX) or with DNA	No recent updates in the clinical setting	
Synthetic lethality	LNA gapmR ASO (MIR17PTi)	No	Upregulation of BIM by targeting miR-17-92s	Preclinical	Morelli *et al*.^[147]^
	PARPi		Addiction to PARP1	Preclinical	Caracciolo *et al*.^[148]^

siRNA: Small interfering RNA; MM: multiple myeloma; MTD: maximum-tolerated dose; PPI: protein-protein interaction.

### Direct inhibition of PPIs or DNA binding

#### Peptidomimetics and other small molecule inhibitors against PPIs

Peptidomimetics are small molecules designed to mimic the binding of a peptide sequence to a target^[149]^. The first inhibitor of the MYC/MAX interaction, ILA6B17, was identified after screening a 7000-molecule peptidomimetic library and suppressed the growth of MYC-transformed chicken embryo fibroblasts (although it was suggested to be slightly unspecific, since it could also inhibit Jun-induced transformation)^[150]^. Other compounds have been identified by multiple different screenings as being more MYC-specific (e.g., 10058-F4, 10074-G5, Mycro3 or KJ-Pyr-9)^[151-154]^. However, there are no recent studies that investigate the therapeutic opportunity of these molecules in hematologic cancers, despite their great promise, leaving the door open for new compounds to be tested.

#### DNA binding inhibitors

Some research groups have instead targeted the binding of MYC, MAX or their dimers, to DNA, using small molecules such as MYRA-A or KSI-3716^[155,156]^ or peptides and mini-proteins, known to have increased selectivity and affinity, as well as lower toxicity. An example of the latter is H1, a 14-amino acid peptide derived from the helix 1 C-terminal region of MYC itself^[157]^. Our group contributed to the effort using the purified Omomyc mini-protein, shifting the use of Omomyc from transgenic construct to a pharmacological tool^[158]^.

The intravenous formulation of Omomyc, OMO-103 is currently being tested in a Phase I/II clinical trial (NCT04808362) for solid tumors. However, its use or the use of similar molecules could be extended to treat blood cancers.

### MYC-dependent synthetic lethality

MYC is a key transcriptional regulator of many genes, including micro-RNAs such as miR-17-92, involved in maintaining cellular homeostasis during MYC-driven tumorigenesis, suppressing the apoptotic program led by the oncoprotein. In this context, Morelli *et al.*^[147] ^hypothesized that inhibition of these miRNAs could be synthetic lethal in MYC-deregulated tumors, such as MM^[147,159]^. The authors used LNA gapmeR antisense oligonucleotides to target all six miR-17-92s. They focused on one particular ASO, MIR17PTi, and demonstrated a robust effect in a relevant preclinical *in vivo* model^[147]^. As for the mechanism underlying the synthetic lethality, the authors noted some resistant cell lines that lacked the expression of the tumor suppressor BIM, speculating that feed-forward loops between MYC and miR-17-92 were required to induce the upregulation of BIM (and other genes) that would trigger apoptosis in this particular context, in which oncogenic MYC controls the apoptotic program^[147]^. Despite the thorough characterization of this new strategy, including PK profiles in non-human primates, a well-defined mechanism of action and impactful antimyeloma effect, to our knowledge, there is no planned clinical study to explore such a compelling alternative yet.

Another attractive investigational area for synthetic lethality is the DNA damage response, as cancer cells are addicted to compensatory DNA repair pathways. Indeed, Caracciolo *et al.*^[148]^ highlighted the potential of using PARP inhibition in MM, in particular in the case of bortezomib resistance, pointing to a new role of MYC in driving PARP-1 mediated repair. Caracciolo *et al.*^[148]^ elegantly explained how MYC contributes to genomic instability, switching the preferential DNA repair mechanisms to the error-prone PARP-mediated alternative non-homologous end joining, and pointed to its potential use as a predictive biomarker for PARPi treatments in MM^[148]^.

## CONCLUSION

In this review, we try to compile the most recent publications describing the use of different MYC inhibitors in hematologic malignancies, especially MM.

The first attempts to use this strategy in the clinic, unfortunately, have not shown much efficacy (INCB054329, GSK126, SY-1365 and DCR-MYC). Nevertheless, a few are proposed to have potential for improved activity when combined with standard antimyeloma therapies (pinometostat, PIM447, leflunomide, selinexor and TAZ-upregulation). For some compounds, we need to report the lack of follow up or publications of recent results in the last years (CX-5461, peptidomimetic inhibitors and other direct MYC inhibitors, such as biologics including peptides and mini-proteins). On a more positive note, however, many of the molecules reviewed here are still being evaluated in clinical trials (RO6870810, IRF4 ASO and AZD4573) or have a strong enough rationale and preclinical evidence to inform the design of clinical trials for their assessment (rocaglates, new BETi, anlotinib, DC-34, D089 and synthetic lethality strategies), giving us some hope that we will eventually see positive clinical outcome.

One aspect that seems clear from most of the studies is that the field of MM has reached a consensus in preferring combinatorial approaches, above all in the relapsed/refractory context. By targeting different aspects of MM cell biology, many of which are ultimately controlled by MYC, the research community expects to achieve higher response rates, delay resistance appearance and resensitize patients who have exhausted all other alternatives, attempting to prolong survival and ultimately getting one step closer to rendering myeloma a curable disease.

## DECLARATIONS

### Copyright

© The Author(s) 2021.
